# Thyroid Cartilage Window Approach to Extract a Foreign Body after Migration into the Paraglottic Space

**DOI:** 10.1155/2018/3590580

**Published:** 2018-03-31

**Authors:** Sheikha Alkhudher, Faisal AlObaid, Shabreez Shafi

**Affiliations:** ^1^Surgical Department, Amiri Hospital, Kuwait City, Kuwait; ^2^ENT Department, Farwaniya Hospital, Sabah Al Nasser, Kuwait

## Abstract

We report a case of fish bone impaction in the paraglottic space, which caused palsy of the left vocal cord. The patient was a 45-year-old man. He presented with throat pain and hoarseness of voice for approximately one week. The diagnosis was made after careful history taking and confirmed by the use of computed tomography scan as the fish bone was not visible endoscopically under local and general anaesthesia. The patient underwent thyroid cartilage window approach, and the fish bone was retrieved. His symptoms have improved significantly, and he did not require tracheostomy. Other cases reported the removal of foreign bodies by other techniques such as laryngofissure and posterolateral approach. Our case is different in that we used a modification of thyroplasty type 1 technique as it has less reported complications than other approaches that were published in literature.

## 1. Introduction

One of the most common clinical problems encountered in the emergency room and otorhinolaryngology outpatient departments is foreign bodies in the digestive tract, especially fish bones in the adult population. However, the impaction of the fish body in the larynx is a rare event and accounts for less than 4% of all foreign bodies [1, 2]. Hence, the migration of the foreign body into the paraglottic space is a rarer event. A review of the literature showed only two cases that reported the migration of an ingested foreign body into the paraglottic space; however, different approaches were used in each case [3, 4]. Usually, the patient presents with pharyngeal pain, odynophagia, chronic cough, neck tenderness, and sometimes, hoarseness. If not treated, it may cause significant complications including deep neck infection, vocal cord palsy, perforation of the oesophagus, retropharyngeal haematoma, and pyopneumothorax, and in rarer cases, it may cause death [5].

In this paper, we describe the case of vocal cord palsy after the migration of fish bone into the paraglottic space that ultimately required surgical intervention for complete removal.

## 2. Case Report

**Figure 1:**
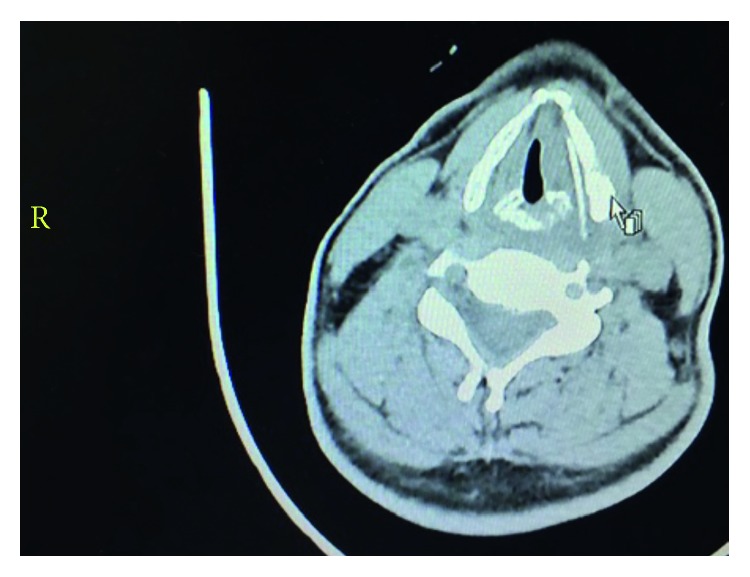
CT scan of the patient's neck.

A 45-year-old man presented to our otorhinolaryngology outpatient department with the symptoms of persistent odynophagia and hoarseness for 1 week after ingesting a fish bone. There was neither dysphagia nor dyspnoea. At first, he sought medical consultation in a local clinic and cervical X-ray was done. However, it was not conclusive, and there was no sign of presence of foreign body in the pharynx. Due to the worsening of his symptoms, he presented to our clinic on 25 August 2016. Flexible pharyngolaryngoscopy was done, and it was noted that there was left vocal cord immobility, oedematous left aryepiglottic fold, and left pyriform fossa with secretions in it. We could not find any foreign body in his pharynx nor in larynx. However, based on his history, the presence of fish bone impaction in the larynx was suspected. The patient was then admitted to the hospital, and urgent computed tomography (CT) scan of the neck was done and revealed a 3 cm linear foreign body embedded in the soft tissue medial to the left thyroid cartilage with surrounding hypodense soft tissue swelling causing mild indentation of the left vocal cord extending into the supraglottic region up to the left pyriform sinus and downwards minimally extending to the infraglottic region causing mild asymmetric narrowing of the involved laryngeal segments and causing mass effect on the glottis and supraglottic airway causing mild asymmetric narrowing (Figure 1).

The diagnosis of inflammation of the paraglottic region and vocal cord immobility secondary to fish bone impaction in the paraglottic space was made. The patient was then shifted to the operation theatre (OT), and rigid direct laryngoscopy was performed under general anaesthesia. It revealed oedema in the left pyriform fossa and left supraglottic region, and no foreign body was visualized. Also, rigid oesophagoscopy was performed, and it showed the same findings as the laryngoscopy. Therefore, foreign body retrieval endoscopically has failed.

**Figure 2:**
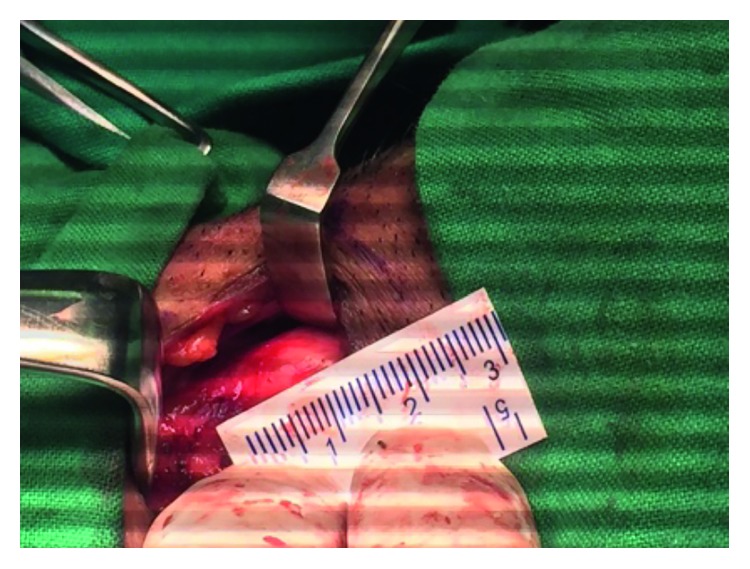
Size of the thyroid cartilage window.

**Figure 3:**
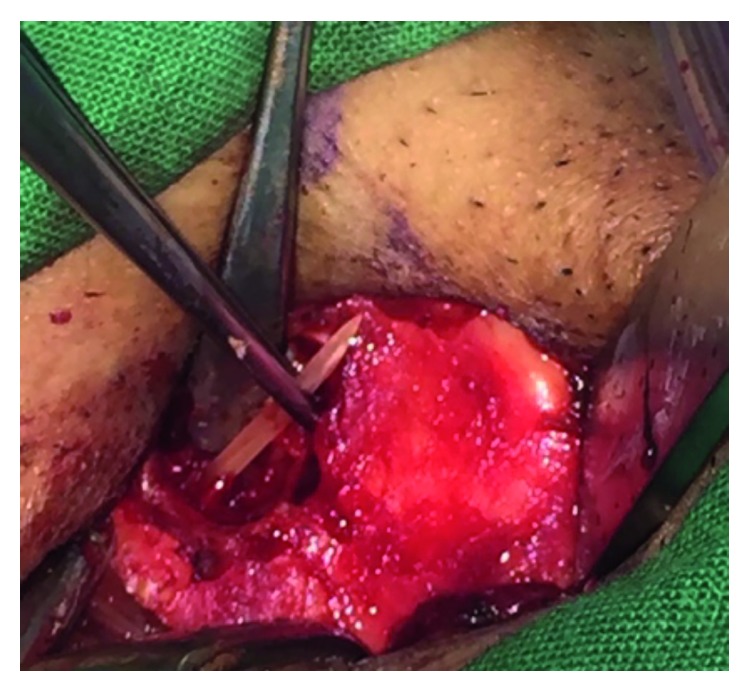
Foreign body was extracted.

Then, we proceeded to the external approach, and thyroid cartilage window with a Skeeter drill window of 12 mm [2] was made and the foreign body was retrieved (Figures 2 and 3). Then, the wound was sutured and a corrugated drain was inserted. The patient was then shifted to the intensive care unit (ICU) for observation, and he was put on mechanical ventilation. We started dexamethasone IV 8 mg TDS to reduce oedema. He was also given Augmentin (amoxicillin/clavulanic acid) IV 1.2 g TDS as a prophylactic, and analgesia was given when required.

The progress of the patient was well, and he was extubated. We started him on oral feeds in the ICU before shifting him to the ward. After his condition improved, he was shifted to the ward and laryngoscopy was repeated, and it showed improvement in the mobility of the left vocal cord and no oedema was noted. In the ward, the patient was started on normal feeds and he tolerated them well and was discharged.

On the following laryngoscopy two weeks later, the swelling of the left pyriform fossa and the left aryepiglottic fold has subsided, and the function of the vocal cords movement was recovered.

## 3. Discussion

Foreign body ingestion into the upper aerodigestive tract is a relatively common clinical problem we face in the emergency room. Among the various objects that can be found, fish bone ingestion is the most common in the adult population [6]. It usually lodges in the palatine tonsil, tongue base, and vallecula. However, there is slight chance of foreign bodies migrating into the pharynx and larynx, but it is considered as a rare event with less than 4% [1, 2]. The symptoms may vary from slight throat discomfort to stridor and hoarseness depending on the site of the obstruction and the size of the foreign body.

As our case showed, plain film might miss a fine fish bone. Therefore, the use of computed tomography (CT) is very helpful in spotting the foreign body in question.

Also, foreign bodies can be often visualized by fiber-optic pharyngolaryngoscopy. In these cases, the retrieval of the foreign body is possible endoscopically or with laryngeal forceps under the guidance of the endoscope [1].

However, foreign body removal cannot be done endoscopically in certain cases and may require more invasive techniques, especially if it was lodged in inaccessible places such as the paraglottic space.

In this paper, we present a case of migration of the fish bone into the paraglottic space. It is quite difficult to know the exact way the fish bone migrated into the paraglottic space. One of the possible ways of its migration is the penetration of the anterior wall of the pyriform sinus. Subsequent contractions of the pharyngeal constriction may have caused the fish bone to propel forward into the paraglottic space as Megwalu's paper proposed [4]. There were two cases published that we found on the removal of the fish bone from the paraglottic space.

Also, Lupo et al. reported a case of ingested bone migrating into the paraglottic space following a maxillofacial trauma [3]. Similar to our case, they were not able to remove the foreign body endoscopically. Therefore, they performed a laryngofissure procedure, and it was successful. The patient then required a tracheostomy and then eventually was decannulated. Megwalu et al. also reported a similar case to ours where the fish bone migrated into the paraglottic space, and after an unsuccessful endoscopic removal of the fish bone, the patient underwent a posterolateral approach to the paraglottic space, which is a modification of the approach used for arytenoid adduction. The patient did not require tracheostomy, and his postoperative course was eventful. So, in this paper, we discuss an alternative approach to the removal of fish bone in the paraglottic space exploration. Our patient underwent a thyroid cartilage window approach, which is a modification approach of the thyroplasty type 1 that is usually used for medialization of the vocal cords. Despite the fact that our patient was intubated, it was only done as precaution postoperatively and for close monitoring. We propose this less morbid approach for the removal of smaller foreign bodies such as fish bone from the paraglottic space as our patient did not require tracheostomy and his postoperative course was uneventful.

## 4. Conclusion

The reason we decided to publish and report this case is to propose an alternative method of extracting small foreign bodies that migrate into the paraglottic space with less morbidity and better outcome.

## Additional Points

*Summary*. (i) Foreign bodies in the paraglottic space can be removed via laryngofissure and posterolateral approach. (ii) Using the listed methods, the patient may require tracheostomy post-op and longer hospital instay and recovery.
